# From Factory to Field: Sex Pheromone of *Plutella xylostella* Produced in Yeast Cell-Factories Validated in Laboratory and Field Trials

**DOI:** 10.3390/insects17030303

**Published:** 2026-03-11

**Authors:** Petri-Christina Betsi, Eleni Koutsoumpeli, Irina Borodina, Dimitris Raptopoulos, Maria Konstantopoulou

**Affiliations:** 1 Chemical Ecology and Natural Products Laboratory, Institute of Biosciences and Applications, NCSR “Demokritos”, 15341 Athens, Greece; betsipetri@bio.demokritos.gr (P.-C.B.); eleni.koutsoumpeli@gmail.com (E.K.); 2 The Novo Nordisk Foundation Center for Biosustainability, Technical University of Denmark, Kemitorvet Building 220, 2800 Kongens Lyngby, Denmark; irbo@biosustain.dtu.dk; 3 Novagrica Hellas S.A., TESPA “Lefkippos”, 15341 Athens, Greece; dimrapto@novagrica.com

**Keywords:** diamondback moth, sex pheromone, yeast-derived pheromones, electroantennographic detection, olfactometer, field monitoring

## Abstract

*Plutella xylostella* (L.) (Lepidoptera: Plutellidae), the diamondback moth (DBM), is a cosmopolitan pest of brassicas. To validate and compare the performance of yeast-derived sex pheromone components with chemically synthesized ones, we studied the behavioral and electrophysiological responses (EAGs) of male DBM adults. In addition, using gas chromatography coupled with electroantennographic detection (GC-EAD), we examined whether any residual impurities present in yeast-derived pheromone components can be perceived by the insects’ antennae and are thus capable of interfering with normal behavior. Furthermore, we assessed the performance of the yeast-derived pheromones under field conditions through monitoring trials conducted in cabbage crops in Greece. Electrophysiological and behavioral assays revealed equivalent responses from the insects to both the yeast-derived (BIO) and chemically synthesized (CHEM) pheromone blends. Consistent with this, GC-EAD results showed no significant differences in antennal response to minor impurities present in the BIO blend compared to the CHEM blend. Finally, it was demonstrated that the binary pheromone blend—comprising (*Z*)-11-hexadecenal and (*Z*)-11-hexadecenyl acetate derived from (*Z*)-11-hexadecen-1-ol produced by yeast cell-factories—was as efficient and specific for trapping male moths in cabbage fields as the conventional ternary synthetic blend [(*Z*)-11-hexadecenal and (*Z*)-11-hexadecenyl acetate and (*Z*)-11-hexadecen-1-ol]. The yeast-derived mixture contained small amounts of unoxidized (*Z*)-11-hexadecen-1-ol due to incomplete oxidation.

## Simple Summary

The use of insect sex pheromones in mating disruption represents a sustainable alternative to conventional insecticides for agricultural pest control. Notably, this method remains effective against insecticide-resistant pest populations. Furthermore, the novel production of pheromones in engineered yeast cell-factories—a cost-effective and innovative platform—is a ground-breaking technology poised to transform modern plant protection strategies. *Plutella xylostella* (Lepidoptera: Plutellidae), the diamondback moth (DBM), is a key pest of oilseed and vegetable crops worldwide and one of the most resistant pest species globally to chemical insecticides. This study demonstrated that in laboratory assays—electrophysiological and behavioral—both the yeast-derived and chemically synthesized pheromone blends elicited equivalent responses from DBM males. In addition, in monitoring trials in cabbage fields in Greece it was confirmed that the DBM binary pheromone blend—comprising (*Z*)-11-hexadecenal and (*Z*)-11-hexadecenyl acetate which were derived from (*Z*)-11-hexadecen-1-ol produced by yeast cell-factories—was as efficient and specific for trapping male moths as the conventional ternary synthetic blend [(*Z*)-11-hexadecenal (*Z*)-11-hexadecenyl acetate and (*Z*)-11-hexadecen-1-ol]. The yeast-derived mixture contained small amounts of unoxidized (*Z*)-11-hexadecen-1-ol due to incomplete oxidation.

## 1. Introduction

The diamondback moth (DBM), *Plutella xylostella* (L.) (Lepidoptera: Plutellidae), is a cosmopolitan pest of oilseed and vegetable crops, particularly cabbage and other cruciferous plants. Under severe infestation, it can cause yield losses of up to 92% in cabbage and 75% in broccoli [1,2,3]. Brassicas are the most widely cultivated vegetables worldwide, occupying 44 million ha and serving a variety of food and industrial uses [4]. Besides brassicas, DBM also attacks some plants from other botanical families [5]. The cost of DBM management has risen, with global estimates now ranging between USD 4 and 5 billion annually [6].

The considerable economic burden of DBM management is attributed to key biological traits of the pest, including its high reproductive rate, short life cycle, multiple generations annually, and broad host range. Its high reproductive rate [7] has led to heavy reliance on insecticides resulting in one of the most resistant pest species globally to organochlorines, organophosphates, carbamates, pyrethroids, to microbial-origin molecules and insect-growth regulators [8,9,10,11,12]. This underscores the urgent need for innovative control methods that are both effective and ecologically sound.

The utilization of sex pheromones is an ideal tool for early monitoring, mass trapping, mating disruption (MD) and push–pull strategies aiming to address the resistance challenges, given their inherent advantages [3,9,13,14,15,16,17]. DBM sex pheromone components were first isolated and identified as (*Z*)-11-hexadecenal (*Z*11-16:Ald) and (*Z*)-11-hexadecenyl acetate (*Z*11-16:OAc) in the 1970s [18,19]. The presence of (*Z*)-11-hexadecen-1-ol (*Z*11-16:OH) in the female’s pheromone gland was also reported [20] and identified as a minor pheromone component [21].

Variations in the composition and relative proportions of DBM sex pheromone components have been reported across different geographic locations [22]. While an 8:2 to 4:6 mixture of *Z*11-16:Ald and *Z*11-16:OAc has been reported as highly attractive to males in the field [23], subsequent studies showed that the addition of *Z*11-16:OH significantly increased bait efficacy. However, the reported optimal concentration for male capture is inconsistent, ranging from 1% [21] to 10% [24]. Other researchers have reported improved trap catches by adding 10% of (*Z*)-9-tetradecen-1-ol (*Z*9-14:OH) to the natural pheromone blend [25]. The synthetic pheromone blend has been utilized to monitor pest populations [26,27], in mass trapping experiments [28], and in an IPM program [29].

The observed differences in DBM trap catches with different ratios of pheromone components may be attributed to genetic variation among populations. This variation can influence the mating behavior in DBM populations from different geographical regions, such that a blend that is highly attractive in one area may be less effective in another. This underscores the necessity of determining the exact pheromone composition of local DBM populations to achieve successful control [22]. It was recently claimed for the first time that the *fruitless* (*fru*) gene might influence the alterations in sex pheromone ratios in female insects [30].

The effective application of DBM sex pheromones for behavioral manipulation—such as monitoring and MD—requires a comprehensive understanding of their underlying physiological and sensory mechanisms. This task is further complicated by the variable blend ratios among different geographical populations of the insect [17,18,31,32,33,34]. This research is critically important in light of a growing market demand for safer, bio-based pesticides, driven by public concern about insecticide residues in food and the adverse impacts of conventional pesticides.

Although pheromone-based management techniques have proven effective, their synthesis represents a considerable cost, rendering them economically non-competitive with conventional insecticides, restricting their use to high-value crops. To lower the cost of pheromone synthesis and promote their wider use in agricultural pest management, biotechnological production of several insect pheromone components has been established in yeasts [35,36,37,38,39] and plants [40,41]. Compared to chemical synthesis, biotechnological production offers several important advantages, such as sustainability, reduced environmental impact, and cost-effectiveness [36].

Using biotechnological methods, various pheromones, including *Z*11-16:Ald, *Z*11-16:OAc and *Z*11-16:OH, were produced using engineered yeast cell-factories. An additional advantage of this biotechnological pheromone production is the capacity to produce both major and minor pheromone components in a single process [35]. Field trials have demonstrated that bio-based pheromones can perform as effectively as their synthetic counterparts. Yeast-derived pheromones for *Helicoverpa armigera*, *Z*11-16:Ald and (*Z*)-9-hexadecenal (*Z*9-16:Ald), proved equivalent to synthetic compounds for both monitoring and mating disruption [42]. Similarly, plant-derived pheromones produced by engineered *Camelina sativa* seeds were as effective as the conventionally synthesized ones for DBM monitoring in cabbage fields [40]. The fermented fatty alcohols were converted to their corresponding aldehydes via oxidation using a tetrakisacetonitrile copper(I) triflate/TEMPO catalyst system [35], while *Z*11-16:OAc was derived from acetylation of *Z*11-16:OH. Notably, the yeast-produced purified broth containing alcohols, after being oxidized into their respective aldehydes, retained about 3% of the starting alcohol (*Z*11-16:OH) as an unoxidized impurity.

In this study, we demonstrate that yeast-derived DBM pheromone components exhibit equivalent efficacy to conventional synthetic pheromones in electroantennogram, behavioral, and field monitoring assays. Validating these yeast-derived components is critical for extending their application for mating disruption beyond high-value cash crops to include widespread row crops.

## 2. Materials and Methods

### 2.1. Insects

A laboratory colony of DBM, originating from wild populations, was established at the Chemical Ecology and Natural Products Laboratory of NCSR “Demokritos”, Athens, Greece. Larvae were reared on fresh cabbage leaves. All life stages were kept at a 16:8 (L:D) photoperiod, at 25 ± 1 °C and 60 ± 5% relative humidity.

### 2.2. Electrophysiological Responses of Male P. xylostella

The antennal responses of male DBM adults to pheromone blends were evaluated by electroantennography (EAG) using a commercially available electroantennographic system (Syntech, Hilversum, The Netherlands). Yeast-derived pheromone constituents, standard compounds, and mixtures of yeast-derived and standard compounds were tested. As standard compounds, the three pheromone components of DBM pheromone, *Z*11-16:Ald, *Z*11-16:OAc and *Z*11-16:OH, chemically synthesized (Bedoukian Research Inc., Danbury, CT, USA with purity > 95%), were used singly or mixed in a 60:30:10 ratio (CHEM). The same compounds produced by engineered yeast cells (Bio-Phero ApS, Copenhagen, Denmark, with 70% overall purity) were used, again, singly or in a 60:30:10 ratio (BIO).

The antenna of a virgin, two-to-three-day-old male adult was mounted on a two-pronged fork probe using a conductive gel (ECG Supergel, CERACARTA, Forlì, FC, Italy). The base of the antenna was connected to the ground, whilst the distal end was connected to the recording electrode. The signal was amplified 10× by a Universal AC/DC pre-amplifier inside the probe and the analog signal was amplified and recorded with a data acquisition controller (IDAC-4, Syntech, Hilversum, The Netherlands).

Standards of chemically synthesized and yeast-derived pheromone compounds, as well as their corresponding mixtures, were dissolved in pentane to provide 100 μg/mL solutions. The mass of yeast-derived compounds was adjusted based on their purity. A 10 μL aliquot of each solution was pipetted onto a piece of filter paper (7 × 30 mm, Whatman no. 1) and the solvent was allowed to evaporate. Next, the paper strip (carrying a 1 μg dose of the test compound) was inserted into a glass Pasteur pipette (~22.5 cm length, ISOLAB, Wertheim, Germany), which was then sealed with polyethylene plug until use.

For stimulus delivery, the tip of the pipette was inserted into a small hole on the wall of a glass tube directed toward the antennal preparation. Stimuli were provided as 0.3 s air puffs into a continuous flow of filtered and humidified air. The air flow, at a 25 cm^3^/s rate, tube diameter 1 cm, was generated by an air stimulus controller (CS-55, Syntech, Hilversum, The Netherlands). At least 1 min was allowed between successive stimulations to let the antenna recover. Control stimuli consisted of a pipette with filter paper and solvent (pentane). A stimulus, consisting of a 1 μg dose of (*Z*)-7-dodecenyl acetate (*Z*7-12:OAc), was provided at regular intervals during each recording session as a reference compound for data normalization. For normalization, the EAG response to each reference stimulus was defined as 100%. Responses to test stimuli were then calculated as a percentage of the average response to the adjacent references. All test compounds and their mixtures were measured on a total of 15 antennal preparations.

Following the 2021 field monitoring trials, a GC-EAD (electroantennographic detection coupled with gas chromatography) was set up by connecting the above-mentioned antennal system to a GC via a heated liner for stimulus delivery. This was done to determine if impurities left in the yeast-derived pheromone blends after the purification process can be perceived by the insect’s antennae and, therefore, have the potential to interfere with its normal sexual behavior. Sample constituents were separated using an Agilent 7890B GC system (Agilent Technologies, Santa Clara, CA, USA) equipped with an HP-5 column (30 m, 0.32 mm, 0.25 μm) and a flame ionization detector (FID). The oven temperature was programmed from 50 °C to 250 °C at a ramp rate of 6 °C/min. The column effluent was split in a 1:1 ratio between the FID and the antennal preparation (EAD). Electroantennographic responses to all test mixtures were recorded from a total of 5 antennal preparations

### 2.3. Behavioral Assays

Behavioral studies were performed in a dark room under low red light (3–5 lux), at 25 ± 1 °C and 60 ± 5% relative humidity, using a glass Y-tube olfactometer (stem: 17 cm, arm: 24 cm each, diameter: 5 cm) connected to a constant airflow of 30 mL/min with the assistance of an air-pump [43]. All tests were conducted during the first two hours of the scotophase [44]. Two hours prior to testing, adult male moths had been transferred in individual 400 mL clear plastic cups covered with perforated lids and left in the conditions of the dark room to acclimate. A volume of 10 μL of the stimulus solution was loaded on a filter paper strip (5 × 15 mm), allowing 30 s for solvent evaporation. A 1 mg/mL pentane solution of each pheromone component or mixture was used to prepare a 10 μg dose, while pentane was used as a blank control. For sample preparation of yeast-derived pheromone compounds, concentrations were adjusted to account for their reported purity. The performance of the yeast-derived pheromone blend (BIO) was compared to the chemical one (CHEM) in laboratory experiments. In both cases, *Z*11-16:Ald, *Z*11-16:OAc, and *Z*11-16:OH were used in a 60:30:10 ratio. The paper strip loaded with pheromone was placed inside one odor arm, while the blank strip was laid in the opposite arm. A two-to-three- day-old adult male insect was released into the olfactometer at the proximal end and its behavior was recorded for 10 min. A positive selection (1st and/or 2nd choice) was recorded when the insect passed beyond 6 cm from the intersection of the Y-tube to any arm.

Finally, the total time spent in each arm (1st and 2nd choice) of the olfactometer was recorded for every insect. After every five behavioral assays, the olfactometer was thoroughly washed with soap and water, rinsed with acetone and dried in the oven at 120 °C. The olfactometer was rotated 180° after each assay to avoid directional bias. For each treatment, 100 males in total were tested.

### 2.4. Monitoring of P. xylostella in the Field

The sex pheromone dispensers used for monitoring male DBM were supplied by Novagrica Hellas SA (Athens, Greece). Adult monitoring was carried out using delta-type traps baited with rubber septa (bromobutyl elastomers) loaded with 200 μg of DBM pheromone, comprising a 60:30:10 ratio of *Z*11-16:Ald, *Z*11-16:OAc and *Z*11-16:OH.

Monitoring trials were carried out in 2021 and 2022 from August to September in Central Greece (Psachna/Euboea island) in three cabbage fields of 2 ha each (location-1: 38°33′34.1″ N 23°36′43.2″ E, location-2: 38°33′37.6″ N 23°37′18.2″ E and location-3: 38°33′30.5″ N 23°37′25.7″ E). In 2021, yeast-produced DBM pheromone components were mixed at proportions similar to those used to prepare control lures from their chemically synthesized counterparts, with amounts adjusted according to their reported purities. In 2022, the yeast-produced pheromone mixture used contained only *Z*11-16:Ald and *Z*11-16:OAc at a 60:30 ratio, while *Z*11-16:OH was not included in the formulation.

A total of 12 Delta-type traps (4 in each plot) were deployed. Each trap was baited either with commercially available pheromone dispensers (CHEM) (Novagrica Hellas SA) or yeast-derived pheromone blend dispensers (BIO), and suspended on wooden poles at a 1.5 m height. Traps were moved 20 m clockwise each week to mitigate positional effects in the fields. Captured males were recorded once per week and removed. Pheromone dispensers were renewed every four weeks.

### 2.5. Data Analysis

Statistical tests were selected according to data distribution, experimental design, and model assumptions to ensure appropriate and reliable inference. The electrophysiological data were subjected to analysis of variance (ANOVA, parametric test) and mean values were separated using Tukey’s honestly significant difference (HSD) test at *p* ≤ 0.05. The Wilcoxon matched pairs test was used to analyze the mean percentage values of the time DBM spent in each arm of the olfactometer (non-parametric test to compare related samples). Chi-square test (SPSS 8.0, SPSS Inc., Chicago, IL, USA) was used to analyze the DBM choices during the olfactometer bioassay (non-parametric test). The weekly trap catch data from 2021 and 2022 for CHEM and BIO pheromone blends were analyzed using the tailed *t*-test at *n*−2 and *p* = 0.01.

## 3. Results

### 3.1. Electrophysiological Responses of Male P. xylostella

**Figure 1:**
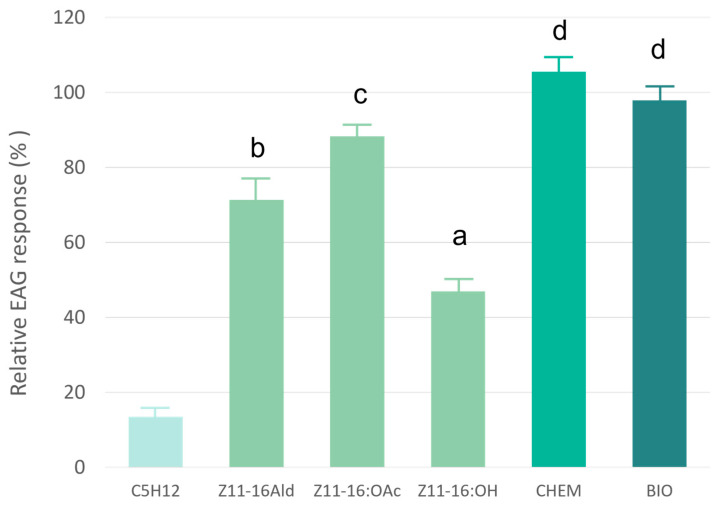
Electrophysiological responses of male DBM antennae to standard compounds (*Z*11-16Ald, *Z*11-16OAc, *Z*11-16OH), to chemically synthesized pheromone blend (CHEM) and yeast-derived pheromone blend (BIO). Means (±SE) followed by the same letter are not significantly different [Tukey’s multiple range tests (HSD) at *p* ≤ 0.05, F = 24.618, df = 4, *p* ≤ 0.001].

In order to evaluate the olfactory perception of pheromone compounds, the antennal responses of *P. xylostella* males to 1 μg of each compound were recorded by EAG. As shown in Figure 1, *Z*11-16:OAc yielded a strong EAG response that was significantly higher than that of *Z*11-16:Ald. *Z*11-16:OH elicited a weaker antennal response. Nonetheless, when these three compounds (*Z*11-16:Ald, *Z*11-16:OAc, *Z*11-16:OH) were presented to the antenna as a mixture, in a 60:30:10 ratio (CHEM), the recorded signal was higher than that of *Z*11-16:OAc alone, suggesting a synergistic effect. The respective mixture of the yeast-derived pheromones (BIO) produced a comparable response (F = 24.618, df = 4, *p* ≤ 0.001) (Figure 1).

**Figure 2:**
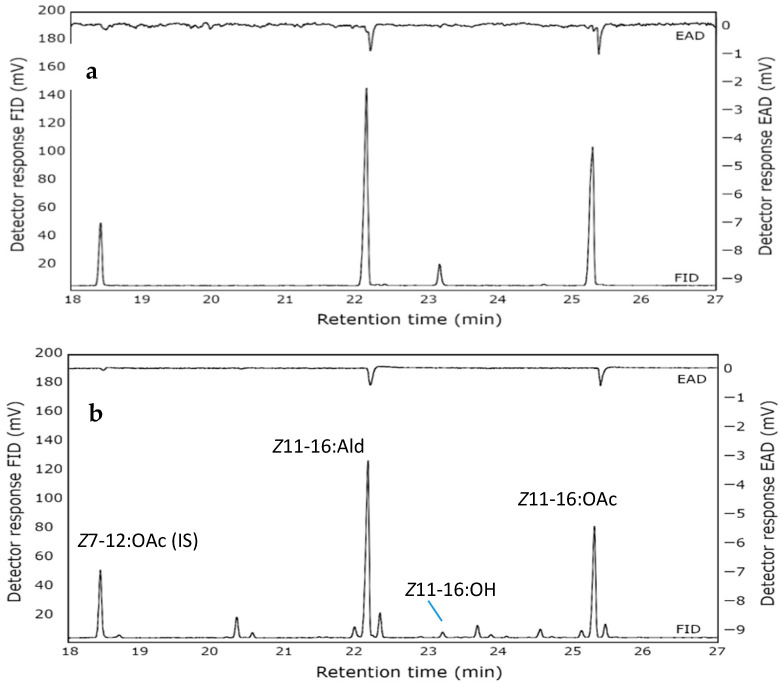
Gas chromatography (GC-FID) linked with electroantennographic detection (EAD) of DBM adults’ antennae to (**a**) chemically synthesized pheromone blend (60:30:10*, Z*11-16:Ald: *Z*11-16:OAc: *Z*11-16:OH; 1 μg) and (**b**) yeast-derived pheromone blend (60:30, *Z*11-16:Ald: *Z*11-16:OAc 1 μg). FID and EAD signals were obtained from three individual GC-EAD runs. *Z*7-12:OAc (200 ng) was added as internal standard.

Since about 3% *Z*11-16:OH was present in the aldehyde derived from the oxidation of the heterologously produced alcohol in engineered yeast, a binary mixture (BIO) consisting of *Z*11-16:Ald and *Z*11-16:OAc (60:30) was prepared for GC-EAD experiments following the 2021 field monitoring trials (Figure 2).

Male DBMs responded similarly to the same composition blends of chemically synthesized and yeast-derived pheromones in EAD. *Z*11-16:Ald and *Z*11-16:OAc elicited strong responses in the male antennae, while *Z*11-16:OH, did not elicit a significant response. *Ζ*11-16:OH was present in the BIO blend and specifically in the yeast-derived *Z*11-16:Ald as a leftover from the conversion of the heterologously produced alcohol to its corresponding aldehyde. No significant antennal responses were recorded for the minor impurities present in the BIO blend.

### 3.2. Behavioral Assays

**Figure 3:**
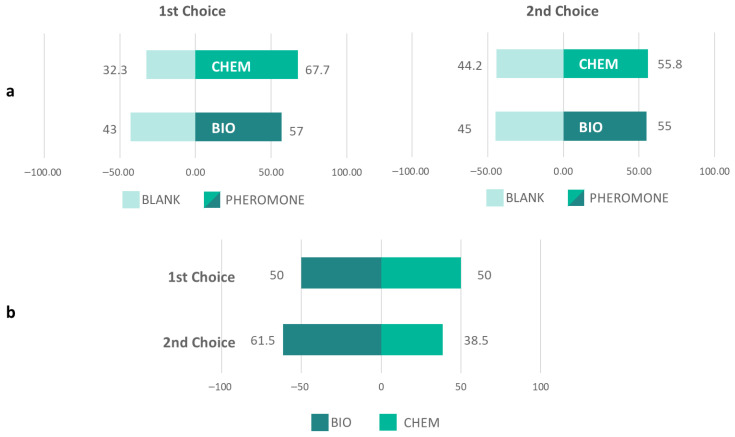
Percentage of DBM males’ behavioral response in Y-tube olfactometer (1st choice, 2nd choice and total) between chemically synthesized (CHEM) and yeast-derived pheromone blends (BIO), and blank (solvent only) (**a**), and between each pheromone blend (CHEM and BIO) (**b**). 3a: [1st choice: CHEM: x^2^ = 16.642, *p* ≤ 0.001, BIO: x^2^ = 4.853, *p* = 0.028], [2nd choice: CHEM: x^2^ = 25.274, *p* ≤ 0.001, BIO: x^2^ = 21.160, *p* ≤ 0.001]. 3b: [1st choice: x^2^ = 0.10, *p* = 1, 2nd choice: x^2^ = 1.385, *p* = 0.239].

In two-choice assays, male moths showed a significant preference for both the CHEM and BIO pheromone blends over the blank control (Figure 3a). The CHEM blend, however, consistently elicited a stronger behavioral response in the first choice (CHEM: x^2^ = 16.642, *p* ≤ 0.001, BIO: x^2^ = 4.853, *p* = 0.028). In the first choice, DBM males exhibited a stronger preference for CHEM compared to blank (CHEM: 67.7%; blank: 32.3%) than for BIO compared to blank (BIO: 57%; blank: 43%). Thirteen and eleven males, respectively, did not make a choice. In the second choice, CHEM (55.8%) and BIO (55%) performed equally well compared to the blank (CHEM: x^2^ = 25.274, *p* ≤ 0.001, BIO: x^2^ = 21.160, *p* ≤ 0.001).

When DBM had to choose between CHEM and BIO pheromone blends, they exhibited the same preference (CHEM: 50%, BIO = 50%) during the first choice (x^2^ = 0.10, *p* = 1) and those individuals that made a second choice showed a preference for CHEM (CHEM: 61.5%, BIO = 38.5%; x^2^ = 1.385, *p* = 0.239) (Figure 3b). Sixteen males did not make a choice.

**Figure 4:**
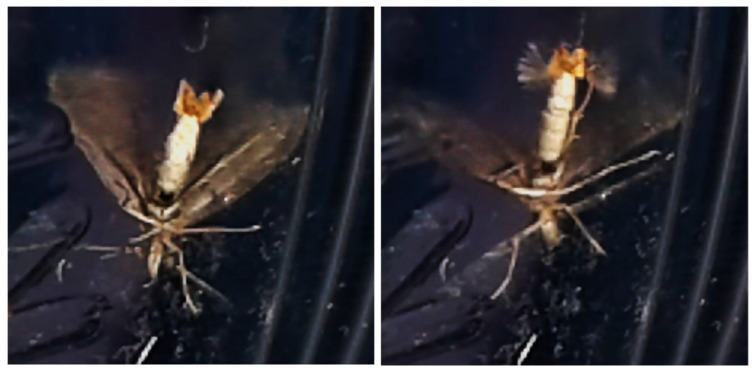
Snapshots of *Plutella xylostella* adult male displaying its hair-pencils while wing fanning.

During the behavioral assays, the characteristic wing-fanning, circling and hair-pencil display of the male courtship behavior were observed when moths were exposed to either the CHEM or BIO pheromone blend (Figure 4).

**Figure 5:**
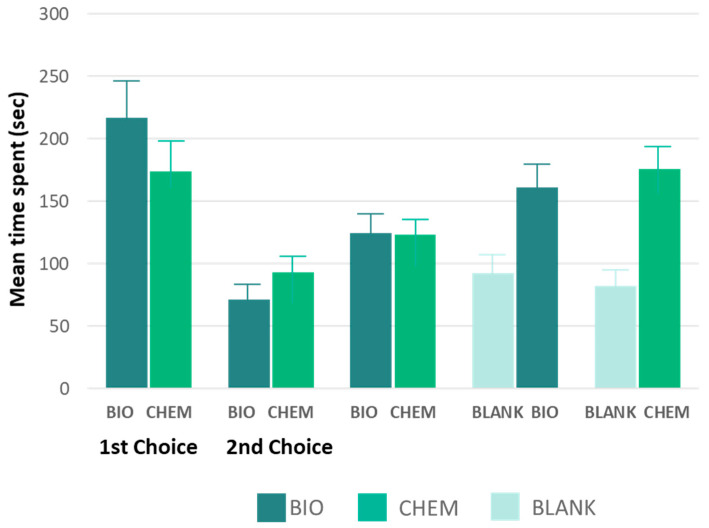
Mean time (sec) spent by DBM males in response to pheromone in Y-tube olfactometer (1st choice, 2nd choice and total) between chemically synthesized (CHEM) and yeast-derived pheromone blends (BIO), and between each blend and the blank (solvent only). [total: z = −0.275 and *p* = 0.783; 1st choice: z = −0.860 and *p* = 0.390; 2nd choice: z = −0.255 and *p* = 0.799], (CHEM: z = −3.602 and *p* ≤ 0.001; BIO: z = −2.592 and *p* = 0.010].

Finally, the time spent in the olfactometer arms (Figure 5) did not vary between the CHEM and BIO pheromone blends (Total: z = −0.275 and *p* = 0.783; 1st choice: z = −0.860 and *p* = 0.390; 2nd choice: z = −0.255 and *p* = 0.799). In contrast, the duration of time spent by DBM males in arms containing pheromone blends (either CHEM or BIO) was significantly longer than that spent in the blank arms (CHEM: z = −3.602 and *p* ≤ 0.001; BIO: z = −2.592 and *p* = 0.010).

### 3.3. Monitoring of P. xylostella Flight in the Field

**Figure 6:**
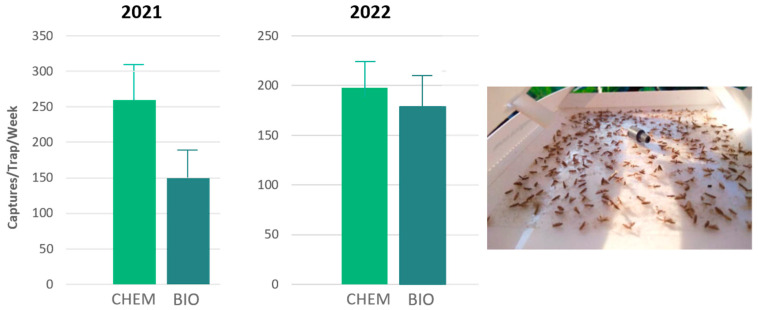
2021 and 2022 DBM weekly trap catches in traps baited with chemically synthesized (CHEM) and yeast-derived (BIO) pheromone blends (**left**). Captures in delta trap (**right**). [2021: (t = 4.705, *p =* 0.001), 2022: t = 1.332, *p* = 0.212].

During the 2021 trials, traps were baited with the standard ternary DBM pheromone blends, prepared in the same ratio from either chemically synthesized (CHEM) or yeast-derived (BIO) compounds with significantly higher catches being obtained in traps baited with the CHEM blend (t = 4.705, *p* = 0.001) (Figure 6).

In the 2022 trials, results showed no statistically significant difference in male catches between traps baited with chemically synthesized pheromone compounds (CHEM) (ternary blend) and those baited with yeast-derived pheromone blend (BIO) in which *Z*11-16:OH was not added (binary blend) (t = 1.332, *p* = 0.212) (Figure 6).

## 4. Discussion

Our electrophysiological results have shown that the standard ternary DBM pheromone blend, produced by yeast cell-factories (BIO), elicited slightly lower, but not significantly so, response from male antennae compared to the blend of chemically synthesized pheromone compounds (CHEM).

In behavioral bioassays, males exhibited no preference between the BIO and CHEM pheromone blends; additionally, the time spent at each pheromone source was also indistinguishable. When presented with either the BIO or CHEM blend versus a blank control, males spent significantly more time at the treated source. Moreover, courtship behavior was the same for both pheromone blends. As documented, females can release sex pheromones to attract males on the day of their emergence. Males use complex odor-producing structures, *coremata* (aka hair-pencils or scent brushes), to release male-produced sex pheromones and then court females by flapping their wings and chasing them [45].

Consistent with previous reports [24], we demonstrated that adding *Z*11-16:OH, to a certain extent, to the binary blend of *Z*11-16:Ald and *Z*11-16:OAc results in an effective lure for monitoring diamondback moth (DBM) populations in the field. In the first year of monitoring trials (2021) the BIO pheromone blend was compared with the CHEM one; both contained all three components. However, the male catches in BIO-baited traps were significantly lower than those in the CHEM-baited traps.

GC-EAD analyses conducted on DBM adults’ antennae following the 2021 field monitoring trials provided a plausible explanation for the reduced trap catches. Consistent with EAG results, GC–EAD showed that *Z*11-16:OH did not elicit a strong antennal response. Antennal responses to the ternary blend of the chemically synthesized pheromone (60:30:10, *Z*11-16:Ald:*Z*11-16:OAc:*Z*11-16:OH) and to the binary blend of yeast-derived pheromone (60:30, *Z*11-16:Ald:*Z*11-16:OAc) provided insight into the observed differences in trap catches. Specifically, no significant antennal responses were recorded for minor impurities present in the yeast-derived pheromone blend (BIO), indicating that suppression of trap catches due to minor impurities in the yeast-derived compounds is unlikely.

In monitoring experiments conducted in 2022, the BIO pheromone blend was reformulated to exclude *Z*11-16:OH. This decision was based on our chemical analyses indicating that the aldehyde component already contained small amounts of unoxidized *Z*11-16:OH due to incomplete oxidation. Consequently, the revised BIO blend consisted only of *Z*11-16:Ald and *Z*11-16:OAc. With this two-component BIO blend formulation, the capture rates of the BIO and CHEM blends were statistically similar.

Our results demonstrate that the yeast-derived pheromone blend consisting of only two components (*Z*11-16:Ald and *Z*11-16:OAc) was just as efficient and specific for trapping of DBM male moths in cabbage fields in Greece as the conventionally produced synthetic pheromone components.

This implies that the residual, about 3%, *Z*11-16:OH present in the aldehyde moiety of the binary blend—which was not converted to its corresponding aldehyde during oxidation of the purified yeast broth—was sufficient to elicit a male behavioral response equivalent to the CHEM blend. Conversely, the addition of extra *Z*11-16:OH in the BIO blend in 2021 raised the total concentration of *Z*11-16:OH to over 13%, leading to reduced trap catch efficacy compared to the CHEM treatment. This indicates a concentration-dependent effect, where a low level of *Z*11-16:OH is permissible or even necessary, but an excess is inhibitory.

Due to its exceptionally high migratory potential, the DBM can be found almost anywhere its host plants grow, and due to climate warming, the harmful activity of this pest is expected to further increase [46]. It can fly 400–500 km in one night and up to 1500 km in a few nights [47], utilizing warm air currents and low-level jet streams [48]. Our field trials were carried out in an area characterized as a “hot spot” for DBM with high infestations. Under these conditions, the BIO pheromone mixture revealed equal activity to the CHEM pheromone mixture in monitoring the pest population.

These results suggest that the yeast-derived pheromones [35] have the potential to attract DBM males. It can be postulated that a blend of the pheromone components produced by yeast cell-factories combined in the precise ratio may be effectively used for the management of the DBM via mating disruption.

Food security and human health depend on the quality and stability of yields in vegetable production [49]. That is the reason why modern agriculture requires insecticides to protect cabbage crops and maximize yields [50].

As the DBM is recognized for its ability to rapidly develop resistance to the majority of chemical classes [51], the use of sex pheromones as a tool for IPM programs rises as a necessity. The biological production of cost-effective sex pheromones is a groundbreaking technology that will enable the rapid expansion of pheromone-based pest management products.

Biotechnological production via microbes or plants offers several advantages over chemical synthesis. First, it utilizes renewable feedstocks like sugars, glycerol, or atmospheric carbon dioxide instead of expensive, fossil-derived chemicals. Second, it simplifies manufacturing by replacing multi-step chemical synthesis—which requires costly catalysts—with streamlined bioconversions, occasionally followed by minimal chemical processing. Third, because these methods employ the insect’s own biosynthetic enzymes, the resulting pheromones often mirror the natural stereoisomer ratios of the target species [36].

In the case of biotechnological pheromones, biosynthesis byproducts that remain in the final product could potentially influence insect behavior. To address this issue, a series of laboratory experiments, including electrophysiological tests (EAG and EAD) and behavioral bioassays, must be performed to confirm the behavioral equivalence of yeast-derived pheromones to chemically synthesized ones. This was demonstrated in *Ostrinia nubilalis*, where the presence of tetradecyl acetate (14:OAc) left in the final product interfered with the insect’s precopulatory behavior [37]. In the current study, our results have shown that minor impurities present in the yeast-derived sex pheromone blend do not affect the behavior of DBM.

We have demonstrated that DBM can be effectively monitored using a two-component yeast-derived pheromone blend [35] consisting of *Z*11-16:OAc and *Z*11-16:Ald, the latter of which contains 3% of its corresponding alcohol. To the best of our knowledge, this is the first reference regarding the successful use of yeast-derived sex pheromone compounds for DBM monitoring. Furthermore, we strongly believe that mating disruption of this pest can be performed using yeast-derived pheromones, as it was demonstrated with *H. armigera* [35,42]. Biological production of low-cost pheromones will enable the rapid adoption of pheromone-based pest management products that can be used as effective, affordable and non-toxic alternatives to insecticides.

## Data Availability

The original contributions presented in this study are included in the article. Further inquiries can be directed to the corresponding author.
